# Book Reviews

**Published:** 1893-08

**Authors:** 


### Book Reviews.

THE ANATOMY AND SURGICAL TREATMENT OF HERNIA. By HENRY O. MARCY, A. M., M. D., LL. D., of Boston. President of the American Medical Association ; Surgeon of the Hospital for Women, Cambridge ; President of the Section of Gynecology, Ninth International Congress ; late President of the American Academy of Medicine ; Member of the British Medical Association ; Member of the Massachusetts Medical Society ; Honorary Member of the New York State Medical Association ; Fellow of the Boston Gynecological Society ; Corresponding Member of the Medico-Chirurgical Society of Bologna, Italy ; Fellow of the American Association of Obstetricians and Gynecologists ; Fellow of the Southern Surgical and Gynecological Association ; late Surgeon U. S. Army, etc. With sixty-six full-page heliotype and lithographic plates, including eight colored plates from Bougery, and thirty-seven illustrations in the text. Sold only by subscripton. Half morocco, $15.00. New York : D. Appleton & Co. 1892.

When one opens a beautiful quarto volume, illustrated with admirable plates, one naturally expects the text to be in keeping with it. Such, however, is not the case with the volume before us. There is a strange medley in this work of quotations from classic authors, proceedings of surgical societies, and a mass of heterogeneous material, all thrown together in a confused, erratic way, which seems to indicate at once that, however skilled a surgeon the author may be, an author he certainly is not. He lacks one most essential characteristic, at least, and that is a proper sense of proportion. From the beginning to the end of the book we find this or that subject given space and exaggerated far beyond its merits, and, at the same time, either scant or no mention of other subjects which certainly deserve some notice, if not an elaborate one. It may be added, incidentally, that the author never forgets himself, and anything which he may have thought or done always gets a prominent place. In parts of the work, this is carried to such an extent that it might fairly be named Dr. Marcy's Treatment of Hernia, instead of having a title indicating a book properly presenting the subject of Hernia with its subdivisions preserving due proportions, and giving a comprehensive *résumé* of the subject.

The author seems remarkably enthusiastic concerning the curative effect of operation. His experience is so different from that of other surgeons, that one wonders whether he takes the pains to keep his cases under observation for a sufficient time to draw correct conclusions. Not a word does he say about danger which he does not offset with some remark which can easily mislead the young surgeon. A fairly large experience in operating for hernia only confirms the belief that, while usually safe, so far as the life of the patient is concerned, the operation itself is attended with many perplexing conditions, upon the complete understanding and mastery over which depends its success. Therefore, we think that any teaching which tends to minimize dangers almost constantly present, is bad teaching for students or youthful surgeons. One could read this book through and never know that fatal hemorrhage could or has occurred, or that the testicle sometimes becomes gangrenous.

Again, almost nothing upon the subject of diagnosis finds a place in the work, and here, also, the book will be of little or no use to those who need it most. No region of the body can furnish more difficult diagnostic problems to the surgeon than the groin sometimes affords, and yet Dr. Marcy passes by this vitally important part of his subject with the scantiest mention. Everywhere, then, throughout the book, the author's lack of sense of proportion is evident. Added to this fault as an author are others equally bad, viz., careless arrangement and doubtful English.

The book contains much good material. It will be useful to the student for the historical information to be found in its pages, and as such it deserves a place with one's books. More than this, however, in all justice, can hardly be said. The author has not accomplished what he set out to do, and the reasons are very evident why he never could succeed. It is a pity that the publishers, who have done their part so perfectly, have not a text in keeping with the beauty and finish of the volume. J. P.

---

DIET FOR THE SICK. By MISS E. HIBBARD, Principal of Nurses' Training School, Grace Hospital, Detroit, and Mrs. Emma Drant, Matron of Michigan College of Medicine Hospital, Detroit. To which has been added Complete Diet Tables for various diseases and conditions, as given by the highest authorities. Detroit, Mich.: The *Illustrated Medical Journal* Co., Publishers. Paper, 74 pages. Price, postpaid, 25 cents; 6 for $1.00.

This little book is a worthy supplement to any cook-book, as it deals only with the dishes suitable for the sick and convalescent; the receipts being favorite ones in use daily in the hospitals wherein the authors are employed. To this has been added the various authorized Diet Tables for use in anemia, Bright's disease, calculus, cancer, chlorosis, cholera infantum, constipation, consumption, diabetes, diarrhea, dyspepsia, fevers, gout, nervous affections, obesity, phthisis, rheumatism, uterine fibroids. It also gives various nutritive enemas. The physician can use it to advantage in explaining his orders for suitable dishes for his patient, leaving the book with the nurse.

---

Hand-Book of Pathological Anatomy and Histology, with an introductory section on Post-Mortem Examinations and the Methods of Preserving and Examining Diseased Tissues. By Francis Delafield, M. D., LL. D., Professor of the Practice of Medicine, College of Physicians and Surgeons, Columbia College, New York, and T. Mitchell Prudden, M. D., Professor of Pathology and Director of the Laboratories of Histology, Pathology, and Bacteriology, College of Physicians and Surgeons, Columbia College, New York. Illustrated by 300 wood engravings printed in black and colors. Fourth revised and enlarged edition. One large octavo volume of 732 pages, with 300 wood engravings, beautifully printed on fine super paper, and bound in blue imported muslin. Price, $6.00. New York: William Wood & Co. 1892.

The authors of this important work have long been before the profession as careful and painstaking writers and investigators. The previous editions of this hand-book have been noticed in the Journal, from time to time, as they have appeared from the press, and we have only to reiterate the favorable judgment we gave upon them and intensify it as applied to this fourth edition, now before us. The authors state that it is intended that the student and practitioner shall find in it the information which they need to enable them to perform autopsies, preserve tissues, and prepare them properly, and to examine them with the microscope. We believe neither the student nor the practitioner will be disappointed in the promise here made. The work comprises instructions in the method of making post-mortem examinations, of preserving its tissues, of preparing them for microscopical examination, and of examining and cultivating bacteria. It also gives the action of the lesions in the different parts of the body, of infectious and general diseases, of violent deaths, and of poisoning; of the changes of inflammation and degeneration, and of the structures of tumors. All these pathological and histological subjects are treated of with a clearness that challenges the admiration of the writers. The work, as heretofore, is well illustrated, and all of the drawings have been made by the authors themselves. This adds very much to their clearness, and elucidates the points of the text, which they aim to elaborate, in an unusual satisfactory manner, while all the color drawings are exceedingly well done. We need not demand an analytical review of the work that has been done so well, and so favorably received by the profession in three previous editions, but desire to commend this fourth and improved edition in an emphatic way. It should be possessed by every one interested in the subjects of which it treats.

---

INTERNATIONAL CLINICS. A Quarterly of Clinical Lectures on Medicine, Neurology, Pediatrics, Surgery, Genito-Urinary Surgery, Gynecology, Ophthalmology, Laryngology, Otology, and Dermatology. By professors and lecturers in the leading medical colleges of the United States, Great Britain, and Canada. Edited by JOHN M. KEATING, M. D., LL. D., Colorado Springs, Col. ; Fellow of College of Physicians, Philadelphia ; formerly Consulting Physician for Diseases of Women to St. Agnes' Hospital ; Gynecologist to St. Joseph's Hospital ; Visiting Obstetrician to the Philadelphia Hospital, and Lecturer on Diseases of Women and Children, Philadelphia ; Editor Cyclopedia of the Diseases of Children. Judson Daland, M. D., Philadelphia ; Instructor in Clinical Medicine, and Lecturer on Physical Diagnosis, and Symptomatology in the University of Pennsylvania ; Assistant Visiting Physician to the University Hospital ; one of the Examiners of the Insane to the Philadelphia Hospital ; Visiting Physician to St. Clement's Hospital, Philadelphia. J. Mitchell Bruce, M. D., F. R. C. P., London, England, Physician and Lecturer on Therapeutics at the Charing Cross Hospital. David W. Finlay, M. D., F. R. C. P., Aberdeen, Scotland, Professor of Practice of Medicine in the University of Aberdeen ; Physician to, and Lecturer on, Clinical Medicine in the Aberdeen Royal Infirmary ; Consulting Physician to the Royal Hospital for Diseases of the Chest, London. Volume I. Third series. Royal octavo, pp. xi.—356. Philadelphia : J. B. Lippincott Co. 1893.

The popularity of this class of periodical literature is attested by the fact that the present year opens with the beginning of the third series of these books. Volume I. is printed and bound in the same form as the preceding numbers of the second and third series, and is a continuation of the same interesting group of literatures. This volume contains a clinical lecture by Dr. Charles G. Stockton, of Buffalo, on Chloride of Gold and Sodium in the Treatment of Senile Fatty Change and Chronic Joint-Affections. This is a new therapeutical application to this intractable class of maladies, and Dr. Stockton's success in the twelve or more cases which he reports is deserving of consideration. He says: "I have seen enough to warrant me in saying that in old lithemic patients quite uniform improvement in all ways apparently follows the use of this medicine continued for several weeks or months." Other contributors to this volume are Dr. Roswell Park, on Brachial Cysts and Bilateral Sciatic Nerve Stretching; Dr. M. D. Mann, on Traumatic (non-septic) Fever following Laparatomy; Dr. Charles Cary, on Tricophytosis Leucoderma, and a case of Syphilis. Among other interesting lectures we may mention one by Dr. William Easterly Ashton, on Nephritic Abscess, Caused by Calculi, and another on Abdominal Section, by Dr. E. E. Montgomery. Whosoever has obtained the preceding volumes will be anxious to add this one to his library.

---

A Hand-Book of Local Therapeutics. General Surgery. By Richard H. Harte, M. D., Demonstrator of Osteology and Syndesmology, University of Pennsylvania; Surgeon to the Episcopal and St. Mary's Hospitals; Consulting Surgeon to St. Timothy's Hospital. Diseases of the Skin. By Arthur Van Horlingen, M. D., Professor of Diseases of the Skin in the Philadelphia Polyclinic and College for Graduates in Medicine; late Clinical Lecturer on Dermatology in Jefferson Medical College; Dermatologist to the Howard Hospital. Diseases of the Ear and Air-passages. By Harrison Allen, M. D., Consulting Physician to the Rush Hospital for Consumption; late Surgeon to the Philadelphia and St. Joseph's Hospitals. Diseases of the Eye. By George C. Harlan, M. D., Surgeon to Wills' Eye Hospital and to the Eye and Ear Department of the Pennsylvania Hospital; Emeritus Professor of Diseases of the Eye, Philadelphia Polyclinic, etc. Edited by Harrison Allen, M. D. Octavo, pp. xxvii.—505. Price, $4. Philadelphia: P. Blakiston, Son & Co., 1012 Walnut street. 1893.

The field of therapeutics has improved its literature to a vast extent within the last decade. General treatises have been revised, enlarged, and brought down to the present, while encyclopedic works have been put forth. This is the first attempt on the part of any author to issue a work on the local action of drugs, and on this account it will command great attention. It must be admitted in favor of this work, that text-books generally are meager in their treatment of the subject, and especially do they omit to mention many agents that specialists find valuable in the treatment of disease. There is a large catalogue of diseases requiring local treatment, and sometimes general management is either unnecessary or subordinate to the local application of agents. On this account, the work before us will prove of value to specialists and teachers, if not to general practitioners. In it, each remedy has been elaborately set forth by different authors, most of whom can boast of great experience in their several departments. One of the useful features of the book is, that each remedy has been taken up in alphabetical order, beginning with the description of its pharmaceutical properties and value, then a consideration relating to its physiological effect and its application and value in local treatment.

We note that some of the drugs heretofore considered of local value have been omitted, while many of the newer drugs have been added to the list of local remedies. The increasing knowledge of asepsis and antisepsis brings out many drugs that otherwise would be assigned to innocuous desuetude, and has relegated others to a second or third place, or wiped them out altogether as remedies. The book is carefully and doubly indexed, one being of remedies and the other of diseases. It is well printed on good paper, and cannot fail to be easily worth the price asked by the publishers.

---

Disease in Children. A manual for students and practitioners. By James Carmichael, M. D., F. R. C. P., Ed., Physical Royal Hospital for Sick Children; University Lecturer on Disease in Children, Edinburgh. Illustrated with thirty-one charts, pp. xvi.—591. Small octavo. New York: D. Appleton & Co. 1893.

It is important that the literature relating to the diseases of children should be kept fresh by the frequent appearances of text-books and manuals, written by competent observers and authors. There is no more important department in medicine than that relating to the diseases of the young, and it ought to be the pride of every physician engaged in general practice to familiarize himself with the manifestations of the maladies of those who are too young to describe their symptoms with intelligence and clearness. These helpless little ones appeal to the sympathy of humanity everywhere, and in no walk of life more than to the physician. One of the most important questions that sanitarians have to deal with is that of school hygiene, and pathology. In other words, the prevention of disease in children is even more important than a knowledge for its cure. Carmichael has very properly begun his talk with a chapter or two on these subjects, and though it is here but briefly discoursed upon, yet they contain elements for much thought, and should stimulate writers into prompt and timely action. The author deals on a comparative extension on disorders of the nervous system to which children are liable. We believe that this is an important branch in the diseases of children, and should be taught with much elaboration. An appendix to the work gives formulæ for the proper preparation of certain foods and medicines, and a useful index furnishes the references given of value.

THE YEAR-BOOK OF TREATMENT FOR 1893. A Critical Review for Practitioners of Medicine and Surgery. A series of contributions by twenty-two writers. In one 12mo volume of 500 pages. Cloth, $1.50. Philadelphia : Lea Brothers & Co. 1893.

For nine years this important annual has appeared, and it is not stating the matter over-strongly to assert that it has improved year by year up to the present time. The improvements in the several departments of medicine and surgery, that have taken place during the past year, are recorded in this book in a most accessible manner and in agreeable form. As we have heretofore said, so we now repeat, that it is one of the most satisfactory year books of treatment that presents itself for the favor of the profession. It is one of the necessary books of reference, and easily finds a place on the book-shelves of every physician, whether general practitioner or specialist.

---

### BOOKS RECEIVED.

Recent Developments in Massage—Historical, Physiological, Medical, and Surgical. By Douglas Graham, M. D., Boston, Mass., Fellow of the Massachusetts Medical Society, Member of Alumni Association of Jefferson Medical College, of the American Medical Association, of the British Medical Association, etc. Physicians' Leisure Library. Second edition ; illustrated. Issued monthly ; price, single copies, twenty-five cents. George S. Davis, Detroit, Mich. 1893.

Electro-Therapeutics of Neurasthenia. By W. F. Robinson, M. D. Physicians' Leisure Library, issued monthly ; subscription price, $2.50 ; single copies, twenty-five cents. George S. Davis, Detroit, Mich. 1893.

Impotence and Sexual Weakness in the Male and Female. By Edward Martin, A. M., M. D., Surgeon to the Howard Hospital ; Clinical Professor of Genito-Urinary Surgery, University of Pennsylvania. Physicians' Leisure Library. Price, single copies, twenty-five cents. George S. Davis, Detroit, Mich. 1893.

Eighteenth Annual Report of the Secretary of the State Board of Health of the State of Michigan, for the fiscal year ending June 30, 1890. Lansing : Robert Smith & Co., State Printers and Binders. 1892.

Cholera : Its Causes, Symptoms, Pathology and Treatment. By Roberts Bartholow, M. D., LL. D., Emeritus Professor of Materia Medica, General Therapeutics, and Hygiene, in the Jefferson Medical College of Philadelphia. In one 12mo volume of 127 pages, with nine engravings. Cloth, $1.25. Philadelphia : Lea Brothers & Company. 1893.

